# Neuropathic-like pain in psoriatic arthritis: evidence of abnormal pain processing

**DOI:** 10.1007/s10067-019-04656-5

**Published:** 2019-07-19

**Authors:** Anoopama Ramjeeawon, Ernest Choy

**Affiliations:** 1 School of Medicine, Cardiff University, Cardiff, UK; 2 CREATE Centre, Section of Rheumatology, Division of Infection and Immunity, Cardiff University, Tenovus Building, Heath Park Campus, Cardiff, CF14 4XN UK

**Keywords:** Anxiety, Depression, Disease activity, Fatigue, Inflammatory arthritis, Neuropathic pain, Pain, PainDetect, Pain severity, Psoriatic arthritis

## Abstract

**Objectives:**

The primary objective was to investigate the prevalence of neuropathic-like pain in patients with psoriatic arthritis (PsA). Secondary outcomes were to investigate whether mood, fatigue, pain, disease severity and fibromyalgia are associated with neuropathic-like pain in PsA patients.

**Methods:**

PsA patients were assessed for fatigue, mood, pain, disease activity and fibromyalgia using questionnaires. Neuropathic-like pain was assessed by PainDetect.

**Results:**

Sixty-four patients with PsA were recruited from the Rheumatology Outpatient Department. Of the 64 patients recruited, 26.6% had ‘likely neuropathic pain’ and 21.9% had ‘possible neuropathic-like pain’ according to the PainDetect questionnaire. Patients with ‘likely neuropathic pain’ had higher disease activity, health assessment questionnaire, patient global self-assessment score, tender and swollen joint counts, dactylitis, enthesitis, pain severity and interference with day-to-day activities, fatigue severity and impact, fibromyalgia, anxiety and depression than ‘unlikely neuropathic pain’ patients (*p* < 0.05). PainDetect score correlated with measures of disease activity, fatigue, depression, anxiety, Widespread Pain Index and Symptom Severity Scale (all *p* < 0.05). Most patients (71%) with neuropathic-like pain fulfilled American College of Rheumatology 2010 fibromyalgia criteria. Patients with ‘possible neuropathic-like pain’ had scores between patients with ‘likely neuropathic pain’ and ‘unlikely neuropathic pain’.

**Conclusion:**

Neuropathic-like pain as evidence of abnormal pain processing is common in patients with PsA. It is associated with higher disease activity and fibromyalgia. A significant proportion of patients had ‘possible neuropathic-like’ pain with intermediate disease and symptom score suggesting neuropathic-like pain as evidence of abnormal pain processing is a continuum rather than concurrent fibromyalgia.

**Table:** 

**Key Points** *• Neuropathic pain is prevalent in psoriatic arthritis.* *• Higher levels of pain, disease activity, fatigue, depression, anxiety and comorbidities in Psoriatic arthritis.* *• Increased pain severity is associated with increased disease activity, fatigue, depression and anxiety.*

## Introduction

Pain is the most common complaint in patients with musculoskeletal diseases. Patients with inflammatory arthritis (IA) such as rheumatoid arthritis (RA) ranked pain as the most important symptom [1], which has a major detrimental impact on their quality of life [2]. Despite advances in treatment for RA, many patients still suffer from pain [3].

Pain can be categorised into inflammatory, nociceptive and neuropathic [4]. Inflammation leads to the release of prostaglandin E2 and prostaglandin I2 which can sensitise nociceptors on sensory neurones to induce pain. In contrast, neuropathic pain is a type of pathological pain [5] and defined as “pain initiated or caused by a primary lesion or dysfunction in the nervous system” [6]. It can affect both the peripheral and central nervous system and may be caused by a variety of pathophysiological mechanisms such as inflammatory reactions and neuroplastic changes [7]. It is characterised by sensory abnormalities ranging from numbness to hypersensitivity (hyperalgesia/allodynia) [8], which can be objectively assessed by quantitative sensory testing (QST) which is a non-invasive method to quantify sensory nerve function, by quantifying the stimulus needed to elicit the perception of specific types of sensation. QST allows for quantification of assessment of perception thresholds for light touch, vibration, thermal and pain sensation [9].

Abnormal pain processing in the central nervous system, central sensitization, has been demonstrated consistently in neuropathic pain [10]. Central sensitization occurs when nociceptive stimulation results in increased excitability of neurons in pain pathways, resulting in pain hypersensitivity, including allodynia and hyperalgesia [5]. Central sensitization has also been demonstrated in rheumatic diseases such as RA [11, 12]. In a previous study investigating the prevalence of neuropathic pain in RA, the most commonly identified neuropathic pain features were ‘pain attacks like electric shocks’ and ‘pain with slight pressure’ [11].

Patients with neuropathic pain typically experience pain that becomes chronic and less responsive to analgesic medication, as well as sleep disturbance, anxiety, depression and reduced quality of life compared with patients with chronic, non-neuropathic pain [13]. In addition to the negative effects in the individual with neuropathic pain, there are also negative effects on society—increased healthcare costs (increased medication needs, increased visits to healthcare providers) and loss of ability to work [14]. Neuropathic pain is in general under-diagnosed and undertreated. Even when diagnosed, it can often be mismanaged—these patients are frequently prescribed conventional analgesics such as non-steroidal anti-inflammatories which have limited efficacy in managing neuropathic pain [15], similarly opiates may not optimally manage neuropathic pain due to resistance or insensitivity [5]. Neuropathic pain is typically treated with antidepressants and anticonvulsants but may also benefit from psychological and occupational therapies to help the patient cope with the pain [5], as even when managed with appropriate pharmacology, benefits may be limited [5, 15]. Understanding the cause, mechanism and symptoms experienced is key to selecting, and future development, of appropriate management of neuropathic pain [5].

The definition of neuropathic pain does not distinguish it from pain arising from neuroplastic changes associated with central sensitization so will be referred to as neuropathic-like pain. Of the inflammatory arthritides, studies to date of neuropathic-like pain have focused on RA; however, psoriatic arthritis (PsA) is another common form of inflammatory arthritis, but neuropathic-like pain and central sensitization have not been assessed in these patients and may impact on disease assessment similarly to RA. The aim of this study is to determine the prevalence of neuropathic-like pain and central sensitization using pressure pain threshold and mechanonociception testing [16]—and PainDetect questionnaire [17] in patients with PsA. The impact of neuropathic-like pain and central sensitization on comorbidities (depression, anxiety) and disease activity assessment will also be assessed.

## Methods

During an eight-week period in 2017, all patients with PsA being treated at the Rheumatology department based at the Cardiff and the Vale University Health Board Trust were invited to take part in the study, either by post or in person by the study faculty when attending routine appointments at rheumatology clinics. Patients were eligible to be included in the study if they had a clinical diagnosis of PsA, were aged eighteen or over and consented to being included in this study. Patients were only excluded if they had phantom limb syndrome or complex regional pain syndrome or did not consent to taking part in the study.

The patients who were enrolled in this study completed a consent form, demographics form (component questions detailed under demographics section of Table 1) and a series of validated questionnaires (Table 1) [18–26], following which they underwent pressure pain threshold and mechanonociception testing and physical examination for enthesitis and dactylitis, on the day of presenting to the unit. The questionnaires were chosen to assess comorbidities and outcome measures that are known to affect pain experience. No follow-up was performed.

**Table 1:** Assessments and questionnaires conducted on each participant in the study

Assessment	Outcome assessed	Primary or secondary outcome
Consent form	Consent	n/a
Demographics form	Demographics (age, sex, disease duration, number of concomitant diseases, number of medications)	n/a
American College of Rheumatology 2010 fibromyalgia criteria [18, 19]	Widespread Pain Index, Symptom Severity Score, fibromyalgia, Polysymptomatic Distress Scale score	Secondary
Brief Pain Inventory [20]	Pain severity, pain interference	Secondary
Fatigue Impact Scale [21]	Fatigue impact	Secondary
Fatigue Severity Scale [22]	Fatigue severity	Secondary
Hospital Anxiety Depression Scale [23]	Anxiety, depression	Secondary
Health Assessment Questionnaire [24]	Disability Index	Secondary
Pain Detect Questionnaire [25]	Pain type	Primary
Patient Disease Activity Assessment Questionnaire [26]	PDAS score, tender joint count, swollen joint count	Secondary
Physical examination	Enthesitis, dactylitis	Secondary
Electronic algometer testing [27]	Pressure pain threshold	Secondary
von Frey filament testing [28]	Mechanical nociceptive threshold	Secondary

The PainDetect questionnaire results classified the patients as ‘unlikely neuropathic’, ‘possible neuropathic’ or ‘neuropathic’ groups. Secondary outcome measures included disease activity assessment, brief pain inventory score, tender and swollen joint count, examination for dactylitis and enthesitis, American College of Rheumatology (ACR) 2010 fibromyalgia (FM) questionnaire score, hospital anxiety and depression scale, fatigue severity score and fatigue impact scale.

Objective assessment of pressure pain threshold and mechanonociception was conducted using an electronic algometer (Somedic Algometer, Somedic SenseLab AB, S*ösdala, Sweden)* [27] and von Frey filaments (North Coast Medical Inc., Morgan Hill, California) [28].

von Frey filament testing was carried out on the mid-point between the wrist and elbow on the anterior surface of the forearms and on the skin over the medial end of the spine of each scapula to elicit the mechanical nociceptive threshold and the mechanical pressure sensation threshold. Electronic algometer testing was carried out at the mid-point of the anterior surface of the thigh and the medial end of the spine of scapulae, to elicit the pressure pain threshold (PPT). For electronic algometer testing and von Frey Filament testing, an average of three measurements taken were recorded—since multiple measurements are needed to reliably estimate the threshold being assessed [16], but considering the time taken for the full assessment for each patient for this study, it was decided to be acceptable to complete three measurements for pragmatic reasons.

The data was analysed using IBM SPSS Statistics for Windows, V23.0. Armonk, NY: IBM Corp. The data was tested for normality using Shapiro-Wilk. The results for all groups at baseline were analysed using ANOVA and post-hoc analysis using Dunnett’s test and then using independent samples *t* test or independent samples Mann-Whitney *U* test as appropriate, to analyse for significant differences. Pearson coefficient correlation testing was carried out to analyse how the parameters assessed varied with ‘pain severity’ and ‘the PainDetect score’.

The study was approved by the South East Coast - Brighton and Sussex Research Ethics Committee (17/LO/0147).

## Results

Sixty-four patients with a clinical diagnosis of PsA, aged > 18 years old, were recruited; their demographics are described in Table 2. From their PainDetect score, seventeen (26.6%) patients were classified into the ‘likely neuropathic pain’ (NP) group, fourteen (21.9%) patients were classified into the ‘possible neuropathic pain’ (PNP) group and 33 (51.6%) patients were classified into the ‘unlikely neuropathic pain’ (UNP) group. In the NP and PNP groups, the majority of patients were female, compared with UNP group, where the majority of patients were male. Disease duration was longest and the number of concomitant diseases was highest in the NP and PNP groups compared with the UNP group, although these differences were not significant (*p* = 0.161 and 0.077). On average, the NP group was taking significantly more medications than the PNP and UNP groups (*p* = 0.025). There were 58 different comorbidities found in the patients enrolled, 15 seen in more than one patient, of which the most common were hypertension (31.0%), type 2 diabetes (13.8%), psoriasis (13.8%) and depression (10.3%).

**Table 2:** Demographics and differences in disease activity, symptoms and comorbidities

	Unlikely neuropathic pain	Possible neuropathic pain	Likely neuropathic pain	*p* value
Demographics				
Number (%)	33 (51.6%)	14 (21.9%)	17 (26.6%)	NS
Age in years, mean (SD)	50.9 (14.9)	49.5 (9.8)	51.4 (16.7)	NS
Female, *N* (%)	12 (36.4)	9 (64.3)	11 (64.7)	NS
Disease duration in years, mean (SD)	10.8 (8.5)	14.4 (15.3)	17.4 (13.7)	NS
Number of concomitant diseases, mean (SD)	1.5 (1.6)	1.6 (1.8)	2.9 (3.1)	NS
Number of medications, mean (SD)	4.5 (2.6)	4.9 (2.9)	7.4 (5.0)*	< 0.05
Assessments mean (SD)				
Disease activities				
Tender joint count	5.2 (5.5)	9.9 (4.8)*	13.2 (7.7)*	< 0.001
Swollen joint count	2.6 (3.7)	6.9 (5.8)*	7.4 (6.8)*	0.003
Patient global assessment score (0–10 cm)	0.92 (0.76)	1.95 (0.90)*	2.29 (1.38)*	< 0.001
Pain severity (0–10 cm)	2.7 (2.2)	4.5 (2.0)*	5.9 (2.0)*	< 0.001
Dactylitis score	1.4 (1.4)	3.8 (3.2)	3.8 (2.0)*	< 0.001
Enthesitis score	1.5 (1.5)	2.2 (1.8)	2.9 (1.7)*	0.015
HAQ score	0.4 (0.5)	1.1 (0.8)*	1.5 (0.8)*	< 0.001
Other symptoms				
Pain interference	2.6 (2.6)	5.0 (2.6)	6.0 (2.0)	< 0.001
Fatigue Impact Scale	29.3 (21.6)	47.6 (13.3)*	55.7 (17.6)*	< 0.001
Fatigue Severity Scale	3.3 (1.8)	5.5 (1.0)*	5.4 (1.7)*	< 0.001
Anxiety	7.1 (4.1)	9.6 (4.7)	10.9 (5.2)*	0.037
Depression	4.5 (4.4)	7.0 (3.4)	9.3 (4.0)*	0.001
ACR2010FM: Widespread Pain Index	2.9 (3.2)	3.4 (2.6)	7.2 (4.5)*	< 0.001
ACRFM2010: Symptom Severity Scale (SSS)	3.8 (2.8)	6.4 (2.3)*	8.1 (2.7)*	< 0.001
CRFM2010: Polysymptomatic Distress Scale (PDS)	6.8 (5.2)	9.8 (4.5)	15.4 (6.3)*	< 0.001
Quantitative sensory testing				
von Frey filaments, mean (SD)	4.61 (0.5)	4.63 (0.4)	4.71 (0.4)	NS
Algometer, mean (SD)	641 (236)	626 (380)	558 (334)	NS

*p < 0.05 when compared with unlikely neuropathic pain group based on Dunnett’s *t* test post ANOVA. *NS*, not significant

Disease activity as measured by tender and swollen joint counts, pain score, patient global assessment, dactylitis and enthesitis scores were statistically significantly higher in the NP than that in the UNP group (Table 2). There were also statistically significant differences in these disease activity assessments in PNP versus UNP except for enthesitis and dactylitis, although for the latter the difference only just failed to reach statistical significance (*p* = 0.057). Across the three groups, disease activity was trending higher over the 3 categories, from unlikely neuropathic pain, to possible neuropathic pain, to likely neuropathic pain [29].

Similarly to disease activity, patients with NP and PNP were statistically significantly more likely have greater pain interference and impact, more severe fatigue and higher levels of anxiety and depression than UNP patients (Table 1).

Using the American College of Rheumatology 2010 fibromyalgia (ACR2010FM) questionnaire to assess “fibromyalgianess” (which can be assessed as a continuum as per the Polysymptomatic Distress Scale score [19]), according to the Widespread Pain Index (WPI), pain was significantly more widespread in the NP group. However, it did not significantly differ between the PNP and UNP groups. Both NP and PNP patients had statistically significantly higher Symptom Severity Scale (SSS), scores than UNP patients. The Polysymptomatic Distress Scale (PDS) scores indicated a significantly greater degree of “fibromyalgianess” in the NP group compared with the UNP group, with no significant difference between the PNP and UNP groups. Twenty patients (5 in UNP, 3 in PNP and 12 in NP) fulfilled ACR2010 criteria for the diagnosis of FM.

Overall PainDetect score correlated positively with measures of disease activity and associated symptoms as well as ACR2010FM PDS score and its components, WPI and SSS (Table 3).

**Table 3:** Pearson correlation between PainDetect score with disease activity and associated symptoms

Assessment parameter	Pearson correlation	*p* value
Widespread Pain Index	0.416	0.001
Symptom Severity Scale (SSS)	0.643	0.000
Polysymptomatic Distress Scale	0.592	0.000
HAQ score	0.624	0.000
Patient assessment score	0.526	0.000
Tender joint count	0.556	0.000
Swollen joint count	0.351	0.005
Pain severity	0.631	0.000
Pain interference	0.620	0.000
Fatigue Impact Scale	0.583	0.000
Fatigue Severity Scale	0.609	0.000
Fibromyalgia	0.509	0.000
Anxiety	0.385	0.002
Depression	0.510	0.002
Dactylitis score	0.508	0.000
Enthesitis score	0.475	0.000
Pressure pain threshold	− 0.320	0.010

Mechanical nociception as assessed by von Frey filaments were similar across 3 groups. PPT evaluated by electronic algometer was lowest in NP patients than that in UNP or PNP patients, but not significantly (Table 1). As PainDetect scores increased, PPT scores decreased (Table 3), indicating an element of central sensitization, a mechanism in the development of neuropathic pain [30].

## Discussion

The results of this study suggest there is a high prevalence of neuropathic-like pain (27%) and possible neuropathic pain (22%) in PsA patients. These patients experience greater levels of pain, and all measures of disease activity were higher. This is similar to a Danish study by Rifbjerg-Madsen et al. [31] investigating the prevalence of neuropathic pain in patients with RA, PsA and other spondyloarthritidies using the PainDetect score, which found a prevalence of neuropathic pain in PsA of 28%, which was significantly higher than the two other types of arthritis investigated. Patients with NP have higher fatigue, anxiety and depression scores. Similar to our results, a large transatlantic study by Taylor et al. [3] in RA patients found that patients with NP having significantly higher pain severity and measures of disease activity. Their study also showed that pain levels were related to fatigue levels and severe pain to be associated with depression.

A limitation of this study may be that our sample population included patients with FM—while only one patient stated they had FM in the demographics questionnaire, according to the ACR 2010 FM criteria [32] there were 20 patients with fibromyalgia (NN 5, PN 3, LNP 12), suggesting that concomitant fibromyalgia is under-diagnosed in patients with PsA. The PainDetect tool has been shown to not be as useful at identifying neuropathic pain in patients with FM [33]. Significantly higher prevalence of FM has been found in PsA patients compared with non-PsA individuals [34], and FM has been found to be associated with worse PsA disease activity [35]. It is also interesting to note that patients with primary FM and psoriasis may be mistaken for patients with PsA due to the similarity in presentation [36]. Patients with neuropathic pain and patients with FM can describe similar sensory perceptions—including burning and prickling pain and allodynia [37]. Not excluding PsA patients with fibromyalgia, or not creating a separate group for such patients, does not permit evaluation of the effect of fibromyalgia on neuropathic pain, for example, whether PsA and fibromyalgia in the same patient may have an additive effect and lead to worse neuropathic pain. Previous studies investigating the prevalence of neuropathic pain, as identified by the PainDetect score, in patients with fibromyalgia and chronic widespread pain found that the majority of these patients have neuropathic pain [38, 39].

Another limitation of this study was the inclusion of patients with type 2 diabetes. Diabetic neuropathy is a common complication of diabetes, which has a varying prevalence depending on the definition of diabetic neuropathy, but affects a greater proportion of diabetics as the time post diagnosis increases [40]. In industrialised countries, diabetic neuropathy is the most common type of neuropathy and neuropathic pain is a common occurrence in length-dependent diabetic neuropathy (diabetic neuropathy with an initial predilection for longer neurons before shorter neurons, affecting 80% of diabetic neuropathy) [41]. Painful diabetic neuropathy has been shown to affect 1/3 of community-based diabetics in a large English population, with having type 2 diabetes being one of the factors of patients with diabetes in whom painful diabetic neuropathy was more prevalent [42]. Diabetic peripheral neuropathy is one of a number of causes of neuropathic pain, which can be detected as probable neuropathic pain using the PainDetect score (44**)** (other causes of neuropathic pain include chronic lower back pain, post-surgical neuropathy, post trauma neuropathy, HIV, trigeminal neuralgia, post-herpetic neuralgia [13, 43]). A previous study found an increased prevalence of diabetes in patients with PsA compared with non-PsA patients and suggested that PsA may have an association with diabetes in females, in particular [44]. Similarly to FM, by not excluding or creating a separate group for the PsA patients with diabetes, it is not possible to assess whether having diabetes has an effect on the neuropathic pain experienced by PsA patients. The demographic data of this study found diabetes was the second most prevalent co-morbidity in the study population.

A study investigating the similarities and differences of diabetic painful neuropathy and FM found that both groups of patients describe their pain as burning/prickling pain with light touch allodynia [37], which has similarities to the characteristics of neuropathic pain described in inflammatory arthritides [11]. While this means that some of the results of the investigations in this study may be secondary to diabetes, in particular type 2 diabetes, and FM, rather than purely PsA, not excluding patients with these conditions makes the study population more similar to the UK population of patients with PsA. This gives our results higher external validity, and an indication that individuals in this population have a prevalence of conditions that may contribute to neuropathic pain.

A key strength of this study was assessing these patients with the ACR2010FM questionnaire. The majority of NP patients (12/17, 71%) fulfilled the criteria for FM. One may interpret our data as evidence of concurrent unrecognised FM in patients with PsA—the prevalence of FM in patients with PsA (20/64, 31%) is much higher than that in the general population of 5% [45]. Furthermore, patients with PNP seem to represent an intermediate “fibromyalgic” phenotype. Only 3 out of 14 patients (21%) fulfilled ACR2010 criteria for FM. Yet their pain severity, disease activity and associated symptoms, WPI and SSS were higher than those of UNP patients. The correlation between PainDetect score with pain severity, measures of disease activity, fatigue, depression, anxiety, WPI, SSS, and PPT strongly suggests a continuum rather than 2 distinct populations of patients. Our interpretation is that “fibromyalgianess” in PsA is a continuum and represents abnormal pain processing in patients experiencing chronic nociceptive pain.

The weaknesses of this study include lack of a control group to compare the study groups with, but as a preliminary study, this is acceptable as the researchers were looking to see if a pattern exists which could be tested more robustly in future. The sample size of this study is relatively small so negative results may be due to a lack of statistical power. Lack of follow-up data meant that the stability of neuropathic pain phenotypes cannot be evaluated. It is also unclear whether more active disease predisposes to NP/fibromyalgia or measures of disease activity are erroneously higher in patients with NP/fibromyalgia.

## Conclusion

There is a high prevalence of neuropathic-like pain in psoriatic arthritis. The causes of this are unknown, and a better understanding of how this develops may help develop better treatments for PsA patients in the future.

PsA patients are severely affected by a variety of symptoms beyond pain and disease activity, which have detrimental biopsychosocial effects. This indicates that their care must be tailored to address all of these problems experienced to achieve the most effective management and therefore best possible quality of life.
